# Qatar Healthcare Workers' COVID-19 Vaccine Hesitancy and Attitudes: A National Cross-Sectional Survey

**DOI:** 10.3389/fpubh.2021.727748

**Published:** 2021-08-25

**Authors:** Rajeev Kumar, Majid Alabdulla, Nahid M. Elhassan, Shuja Mohd Reagu

**Affiliations:** ^1^Department of Psychiatry, Hamad Medical Corporation, Doha, Qatar; ^2^College of Medicine, Qatar University, Doha, Qatar; ^3^Weill Cornell Medicine- Qatar, Doha, Qatar

**Keywords:** COVID-19 vaccine, hesitancy, healthcare workers, safety, efficacy, VAX scale

## Abstract

**Introduction:** Healthcare workers are the critical frontline workforce of the COVD-19 pandemic and are considered a target group for vaccination. Hesitancy to vaccinate is a major concern that can jeopardize the vaccination programme. The hesitancy rates in the general population and healthcare workers (HCWs) vary globally, and more importantly, hesitancy in HCWs is of particular concern, as it can influence the wider population.

**Materials and Methods:** The present study evaluated the vaccine hesitancy rate and its sociodemographic and attitudinal factors among the HCWs in the state of Qatar. We conducted a national cross-sectional survey using a validated hesitancy measurement tool between October 15 and November 15, 2020. A total of 7,821 adults above the age of 18 years out of the 2.3 million adult Qatari residents completed the survey. While majority of the participants were from the general public, 1,546 participants were HCWs. Sociodemographic data, along with attitudes and beliefs around COVID-19 vaccination, were collected from the respondents.

**Results:** We found that 12.9% of the study participants showed vaccine hesitancy, defined as definitely or probably will not take the vaccine if offered, and 25.31% reported that they were unsure about the uptake of the COVID-19 vaccine. Female respondents were more hesitant toward the vaccine. Safety and efficacy concerns of vaccine were the significant predictors of vaccine hesitancy. The primary predictor for vaccine acceptance was a better understanding of the disease and vaccine.

**Discussion:** Overall, 1 in 8 HCWs were reluctant to get vaccinated against COVID-19, mainly due to concerns about the vaccine's efficacy and safety. Education about the vaccine's safety and efficacy can potentially improve acceptance among healthcare workers.

## Highlights

- This was the first study to report vaccine hesitancy in healthcare workers in Qatar, a country with the majority of the population being migrants.
- Vaccine hesitancy in HCWs was 12.9%, and it was much less compared to some other studies globally.
- The main predictors for vaccine hesitancy were female gender and concerns about the vaccine's safety and efficacy.

## Introduction

Coronavirus disease 2019 (COVID-19) is a highly contagious infectious disease caused by the Severe Acute Respiratory Syndrome Coronavirus 2 (SARS-CoV-2) that has transformed the world into a state never witnessed in our lifetime. In the absence of any valid treatment, globally, many countries have imposed strict preventive control measures to restrict the COVID-19 outbreak by implementing social distancing and compulsory use of face mask (1). Further, the development and distribution of vaccines have been one of the cardinal preventive strategies to lessen the spread of COVID-19 (2).

After one of the fastest vaccine development processes by countries and pharmaceutical companies worldwide, currently, the vaccines are available globally, including in Qatar. The Qatar Ministry of Public Health approved Pfizer BioNTech and Moderna COVID-19 vaccines, and the early recipients of vaccines were healthcare professionals, elderly patients and individuals affected with chronic and autoimmune disorders. However, the successful outcome of any vaccination strategy mainly relies on a high vaccine acceptance rate (3). Therefore, vaccine hesitancy is challenging for healthcare professionals and the Ministry of health to build confidence in an emergency-released vaccine rollout to the public.

Vaccine hesitancy is defined as “the delay in acceptance or refusal of vaccination despite the availability of vaccination services.” Further, it is not an all or none phenomenon, which means, some will definitely not take a Covid-19 vaccine if offered, whereas others are unsure of their intention to get vaccinated. Vaccine hesitancy is a global concern, and it is one of the crucial factors of under-vaccination (4). WHO in 2019 stated that vaccine hesitancy is one of the top ten global health threats and it acts as a significant barrier to the success of the immunization programs (5). Interestingly, negative information about the vaccine propagated in some social media platforms might also have been contributing to vaccine hesitancy (6).

Earlier studies reported that vaccine hesitancy was a major concern worldwide with a wide range of reasons for vaccine refusal (7, 8). The most common reasons for vaccine hesitancy, in general, have been perceived risks vs. benefits of vaccine, religious considerations, and most importantly, lack of awareness and knowledge (9–11). To a great extent, these factors are also applicable for COVID-19 vaccine hesitancy in general public and HCWs. In this regard, recent studies demonstrated a significant correlation between willingness to receive COVID-19 and its safety outcome (12), negative attitude and unwillingness to receive COVID-19 vaccines (13), and, importantly, the effect of religious belief and decreased intention to receive COVID-19 vaccines (14). Further, willingness to accept vaccines is governed by various other factors such as cognitive, psychological, sociodemographic and cultural that can also influence individual response to vaccine hesitancy (15).

Healthcare workers (HCWs) orchestrate a vital role in the success of immunization programs. Studies indicated that their knowledge and attitude toward vaccines play an important role in the success of any immunization programs. It has been shown that their knowledge and attitudes about vaccines govern their own vaccine uptake intentions and recommendations to the wider general populations (16, 17). Emerging reports indicate that vaccine hesitancy in HCWs can negatively impact on vaccine acceptance in the general population (18, 19). HCWs who elicit negative attitudes are hesitant toward own vaccinations, and more importantly, they can amplify these undesirable perceptions resulting in even poorer uptake of vaccinations in their patients (20).

A recent wider population-based study of the adult Qatar general population displayed an overall vaccine hesitancy of 20% toward the COVID-19 vaccine (21). The present report specifically explores the hesitancy rates and attitudes among Qatar's HCWs toward COVID-19 vaccination.

## Materials and Methods

### Study Design

We conducted a national cross-sectional survey in Qatar between October 15 and November 15, 2020, using an online survey among the HCWs. The survey link was posted online and advertised through local newspapers and various social media platforms of the Hamad Medical Corporation, the state-funded primary healthcare provider. The advertisements were accompanied by short videos in English and Arabic explaining the survey's rationale and nature. The survey was available in both English and Arabic languages.

### Participants

In the primary survey, all 2.3 million adult residents of Qatar were eligible. A total of 7,821 adults completed the survey. Among

those, there were 1,546 HCWs, and the rest were general public. This report included 1,546 HCWs, 18 years of age and above, who consented to participate.

### Study Materials

A validated vaccine hesitancy measurement tool, The Vaccine Attitudes Examination Scale (VAX) (22) was used as part of a composite questionnaire to assess the vaccine attitudes, awareness, and hesitancy among the study participants. This tool was translated into Arabic, and validation of the translated version was carried out using the guideline published by Sousa et al. (23). The survey also collected relevant demographic and contextual information of the participants.

### Outcome Measures

The selection of study tools (VAX) and the composite questionnaire design were guided by the SAGE group recommendations to assess vaccine hesitancy. These included contextual factors such as ethnic origin, gender, socioeconomic status, educational level, media impact, and individual perception of the pharmaceutical industry; individual and group influences such as previous vaccination experience, beliefs and attitudes to vaccination in general, knowledge and awareness of the COVID-19 pandemic and vaccines, trust in health systems, and perception of risk and benefits of vaccines; and vaccine specific issues such as risks of a new vaccine, risk to children and older adults, and healthcare professionals' role.

### Data Analysis

Descriptive statistics and multivariable logistic regression analysis were done using SPSS version 25.

## Results

### Demographics

The total numbers of respondents of the study were 1,546. Forty two percent of the respondents were in the age group between 26–35 years, followed by 32.6% in the age group between 36–45 years. Further, 56.5% of respondents were males, and 81.6% were married. The majority of the respondents were Asians (52.5%), followed by Arab non-Qatari (24.4%) and Qatari (5.4%). Seventy nine percent were university graduates, and the majority of them were salaried employees (79.3%). The data are shown in Table 1. We did not ask participants to identify their professions to improve the response rate and respecting their privacy.

**Table 1:** Demographic data and characteristics of participants (*n* = 1546).

**Variables**	**Frequency (n)**	**Percentage (%)**
Age group		
18–25	27	1.7
26–35	661	42.8
36–45	504	32.6
46–55	228	14.7
56–65	100	6.5
>65	26	1.7
Gender		
Male	672	43.5
Female	874	56.5
Nationality		
Qatari	83	5.4
Arab-non-Qatari	377	24.4
Asian	807	52.2
African	194	12.5
European	61	3.9
North-America	18	1.2
Central America	4	0.3
South America	2	0.1
Education		
High school degree	87	5.6
Trade/vocational training	29	1.9
University	1,226	79.3
Others	204	13.2
Occupation		
Salaried	1,456	94.2
Self employed	39	2.5
Unemployed	24	1.6
Retired	27	1.7
Marital status		
Single	285	18.4
Married	1,261	81.6
Are you pregnant or breast feeding?
Pregnant	22	2.5
Breast feeding	75	8.7
N/A	769	88.8
How many members/individuals living with you?		
Median (IQR)	4.00 (3.00)	

### Intention to Accept the Vaccine, Health Conditions, Worries About COVID-19, and General Attitude Toward Vaccination

In response to the question, “Will you take the COVID-19 vaccine when it becomes available?”, 61.81% responded that they would “probably or definitely” accept the vaccine. Twenty five percent were unsure, and 12.9% responded that they would “probably or definitely” not take the vaccine.

Almost 95% received childhood vaccination, 60.7% received influenza vaccine annually, 78% revealed no history of any illness, 40.38% had hypertension, and 1.9% had mental health illness. The major worries were family members getting infected (62.0 %) or individuals getting infected (45.3%). In response to the question, “why you are willing to take the vaccine?”, 82.5% responded that they have a good understanding of the disease and the vaccination. Forty four percent of respondents reported that they would recommend the vaccine to others, 23.8% were unsure, and 20.8% reported probably they would take the vaccine. For the question, “Will you get your children vaccinated?”, 39.5% responded definitely, 23.4% were unsure, and 20% reported as probably. For the question, “Do you prefer to go quarantine during traveling or take the vaccine?, 49.3% responded that they would definitely take the vaccine during traveling, 29.2% reported probably, and 21.5% were unsure. For the question, “Do you have any chronic medical conditions or are you taking any long-term medications”, 61.6% reported that they had chronic condition. Only 11.7% had any chronic medical condition/s in the vaccine hesitancy group. The data are shown in Table 2.

**Table 2:** Intention to accept vaccine, health conditions, worries about COVID-19, and general attitude toward vaccination.

**Questions**	**Frequency (n)**	**Percentage (%)**
Will you take the COVID-19 vaccine when it becomes available?		
Definitely	619	43.9
Probably	255	18.0
Not sure	358	25.3
Probably not	92	6.5
Definitely not	90	6.4
Total	1,414	100.0
Have you completed your childhood vaccination?		
Yes	1,395	94.9
No	75	5.1
Total	1,470	100.0
How often you receive the influenza vaccine		
Annually	893	60.7
Twice	176	12.0
Once	190	12.9
Never	211	14.4
Total	1,470	100.0
Do you have any medical illness?		
Yes	324	22.0
No	1,146	78.0
Total	1,470	100.0
Chronic illnesses		
DM	120	32.97
HTN	147	40.38
Dyslipidaemia	43	11.81
Asthma	44	12.09
IHD	10	2.75
Total	364	100.0
Do you have mental health illness?		
Yes	27	1.9
No	1,431	98.1
Total	1,458	1.9
Do you have any psychiatric disorders		
Depression	13	40.63
Anxiety	17	53.12
Bipolar	2	6.25
Total	32	100
Do you have chronic medications?		
Yes	427	30.2
No	987	69.8
Total	1,414	100
Have you or family member had COVID-19?		
I have had COVID-19	71	5.0
A family member has had COVID-19	84	5.9
I and at least one family member has had covid-19	61	4.3
Neither me nor them	1,198	84.7
Total	1,414	100.0
What are you most worried about during COVID-19?		
Fear of becoming infected	700	45.27%
Fear of a family member to be infected	962	62.2%
Financial worries	325	21.0
Job related worries	444	28.7
No available vaccine	539	34.9
Somewhat worried	265	17.1
No worries	159	10.3
Total	1,546	100.0
Why you are willing to take the vaccine?		
My understanding of the disease and the vaccination	714	82.5
Information from my doctor/hospital	81	9.4
Information from social media	30	3.5
Information from news	37	4.3
Information from family friend	3	0.3
Total	865	100.0
Will you recommend the vaccine?		
Definitely	613	44.0
probably	289	20.8
not sure	331	23.8
probably not	82	5.9
Definitely not	77	5.5
Total	1,392	100.0
Will you get your children vaccinated?		
Definitely	550	39.5
probably	278	20.0
not sure	326	23.4
probably not	131	9.4
definitely not	107	7.7
Total	1,392	100.0
Do you prefer to go Quarantine during traveling or take the vaccine?		
I would definitely take the vaccine	627	49.3
I would probably take the vaccine	372	29.2
Not sure	274	21.5
Total	1,273	100.0

### VAX Scale of Hesitancy

**Figure 1:**
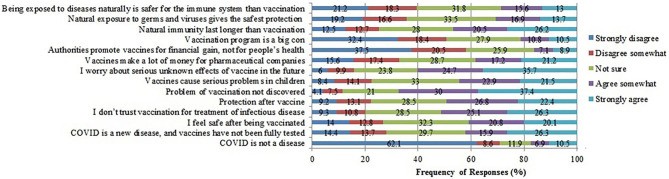
Beliefs toward COVID-19 vaccine and immunity.

There were 14 questions in the VAX scale of hesitancy. The questions included “COVID is not a disease, COVID is a new disease and vaccines have not been fully tested, I feel safe after being vaccinated, I don't trust vaccination for treatment of infectious disease, I feel protected after vaccination, Problems of vaccination not yet discovered, Vaccines cause serious problems in children, I worry about serious unknown effects of vaccines in the future, Vaccines make a lot of money for pharmaceutical companies, Authorities promote vaccines for financial gain and not for peoples' health, Vaccination program is a big con, Natural immunity lasts longer than vaccination, Natural exposure to germs and viruses gives the safest protection, and Being exposed to diseases naturally is safer for the immune system than vaccination”. The questions were rated as strongly agree, agree somewhat, not sure, disagree somewhat, and strongly disagree. The data are shown in Figure 1.

### Association Between Demographics, Variables Related to Attitude and Perception of Vaccination With Vaccine Hesitators vs. Non-hesitators

We divided the responders into vaccine hesitators and vaccine non-hesitators, and their associations with sociodemographic variables and variables related to attitude and perception regarding vaccination were analyzed. Several sociodemographic factors were significantly associated with vaccine hesitancy, including age, nationality, marital status, and gender.

The other significant factors related to attitude and perception were influenza vaccination history, recommendation of vaccine to children, preference of quarantine over the vaccine, the perception that COVID-19 is not a disease, COVID-19 vaccine is not safe, no protection from the vaccine, lack of trust in the vaccine's efficacy, lack of trust of pharmaceutical companies and their intentions, and a belief that natural immunity is better than vaccination. The univariate analysis results are shown in Table 3.

**Table 3:** Univariate analysis of demographics, variables related to attitude and perception of vaccination, comparing vaccine hesitators and non-hesitators.

**Variables**	**Will you have the vaccine when it becomes available?**		**Total*n* (%)**	***p*-value**
	**YES**	**NO**		
	**Vaccine non-hesitators *(definitely + probably) n* (%)**	**Vaccine hesitators *(definitely not + probably not)n* (%)**		
Age				
from 18–25	20 (87.0)	3 (13.0)	23 (100.0)	0.027
from 26–35	550 (90.0)	61 (10.0)	611 (100.0)	
from 36–45	390 (83.7)	76 (16.3)	466 (100.0)	
from46–55	185 (88.5)	24 (11.5)	209 (100.0)	
from 56–65	69 (81.2)	16 (18.8)	85 (100.0)	
>65	2 (10.0)	18 (90.0)	20 (100.0)	
Total	1,232 (87.1)	182 (12.9)	1,414 (100.0)	
Nationality				
Qatari	49 (71.0)	20 (29.0)	69 (100.0)	0.001
Arab-non- Qatari	279 (78.6)	76 (21.4)	355 (100.0)	
Asian	682(92.8)	53 (7.2)	735 (100.0)	
African	159 (91.4)	15 (8.6)	174 (100.0)	
European	46 (79.3)	12(20.7)	58 (100.0)	
N. America	11 (64.7)	6 (35.3)	17 (100.0)	
C. America	4 (100.0)	0 (0.0)	4 (100.0)	
S. America	2 (100.0)	0 (0.0)	2 (100.0)	
Total	1,232 (87.1)	182 (12.9)	1,414 (100.0)	
Marital status				0.014
Single	212 (82.5)	45 (17.5)	257 (100.0)	
Married	1,020 (88.2)	137 (11.8)	1,157 (100.0)	
Total	1,232 (87.1)	182 (12.9)	1,414 (100.0)	
Gender				0.026
Male	541 (89.4)	64 (10.6)	605 (100.0)	
Female	691 (85.4)	118 (14.6)	809 (100.0)	
Total	1,232 (87.1)	182 (12.9)	1,414 (100.0)	
Influenza vaccination				
Annually	792 (92.3)	66 (7.7)	858 (100.0)	0.001
Twice	146 (84.4)	27 (15.6)	173 (100.0)	
Once	147 (81.2)	34 (18.8)	181 (100.0)	
Never	147(72.8)	55(27.2)	202 (100.0)	
Total	1,232 (87.1)	182 (12.9)	1,414 (100.0)	
Will you recommend the vaccine to your children?	546 (99.3)	4 (0.7)	550 (100.0)	0.001
Definitely	277 (99.6)	1 (0.4)	278 (100.0)	
Probably	305 (93.6)	21 (6.4)	326 (100.0)	
Not sure	64 (48.9)	67 (51.1)	131 (100.0)	
Probably not	18 (16.8)	89 (83.2)	107 (100.0)	
Definitely not	1,210 (86.9)	182 (13.1)	1,392 (100.0)	
Prefer quarantine over vaccine				0.001
Definitely will take the vaccine	621 (99.0)	6 (1.0)	627 (100.0)	
Probably will take the vaccine	354 (95.2)	18 (4.8)	372 (100.0)	
Not sure	129 (47.1)	145 (52.9)	274 (100.0)	
Total	1,104 (86.7)	169 (13.3)	1,273 (100.0)	
COVID-19 is not a disease	718 (90.9)	72 (9.1)	790 (100.0)	0.001
Strongly disagree	90 (81.8)	20 (18.2)	110 (100.0)	
Somewhat disagree	121 (80.1)	30 (19.9)	151 (100.0)	
Not sure	72 (81.8)	16 (18.2)	88 (100.0)	
Somewhat agree	103 (76.9)	31 (23.1)	134 (100.0)	
Strongly agree	1,104 (86.7)	169 (13.3)	1,273 (100.0)	
COVID-19 vaccine will not be safe				
Strongly disagree	176 (96.2)	7 (3.8)	183 (100.0)	0.001
Somewhat disagree	169 (96.6)	6 (3.4)	175 (100.0)	
Not sure	363 (96.0)	15 (4.0)	378 (100.0)	
Somewhat agree	179 (88.6)	23 (11.4)	202 (100.0)	
Strongly agree	217(64.8)	118 (35.2)	335 (100.0)	
Total	1,104 (86.7)	169 (13.3)	1,273 (100.0)	
I feel safe after vaccination				
Strongly disagree	88 (49.4)	90 (50.6)	178 (100.0)	0.001
Somewhat disagree	126 (77.3)	37 (22.7)	163 (100.0)	
Not sure	383 (93.2%	28 (6.8)	411 (100.0)	
Somewhat agree	258 (97.4)	7 (2.6)	265 (100.0)	
Strongly agree	249 (97.3)	7 (2.7)	256 (100.0)	
Total	1,104 (86.7)	169 (13.3)	1,273 (100.0)	
I don't trust vaccine to manage pandemics				
Strongly disagree	76 (64.4)	42 (35.6)	118 (100.0)	0.001
Somewhat disagree	103 (74.6)	35 (25.4)	138 (100.0)	
Not sure	317 (87.3)	46 (12.7)	363 (100.0)	
Somewhat agree	295 (92.5)	24 (7.5)	319 (100.0)	
Strongly agree	313 (93.4)	22 (6.6)	335 (100.0)	
Total	1,104 (86.7)	169 (13.3)	1,273 (100.0)	
I will feel protected after vaccination				
Strongly disagree	61 (52.1)	56 (47.9)	117 (100.0)	0.001
Somewhat disagree	126 (75.4)	41 (24.6)	167 (100.0)	
Not sure	322(88.7)	41 (11.3)	363 (100.0)	
Somewhat agree	325 (95.3)	16 (4.7)	341 (100.0)	
Strongly agree	270 (94.7)	15 (5.3)	285 (100.0)	
Total	1,104 (86.7)	169 (13.3)	1,273 (100.0)	
Vaccination is for financial gain				
Strongly disagree	449 (93.9)	29 (6.1)	478 (100.0)	0.001
Somewhat disagree	223 (85.4)	38 (14.6)	261(100.0)	
Not sure	288 (87.3)	42 (12.7)	330 (100.0)	
Somewhat agree	71(78.0)	20 (22.0)	91 (100.0%	
Strongly agree	73 (64.6)	40 (35.4)	113 (100.0)	
Total	1,104(86.7)	169 (13.3)	1,273 (100.0)	
Vaccination programs are big Con				
Strongly disagree	375 (91.0)	37 (9.0)	412(100.0)	0.004
Somewhat disagree	198 (84.6)	36 (15.4)	234 (100.0)	
Not sure	309 (87.0)	46 (13.0)	355 (100.0)	
Somewhat agree	116 (84.1)	22(15.9)	138 (100.0)	
Strongly agree	106 (79.1)	28 (20.9)	134 (100.0)	
Total	1,104 (86.7)	169 (13.3)	1,273 (100.0)	
Natural immunity last longer than vaccinations				
Strongly disagree	147 (92.5)	12 (7.5)	159 (100.0)	0.001
Somewhat disagree	153 (94.4)	9 (5.6)	162 (100.0)	
Not sure	316 (88.5)	41(11.5)	357 (100.0)	
Somewhat agree	227 (87.0)	34 (13.0)	261(100.0)	
Strongly agree	261 (78.1)	73 (21.9)	334 (100.0)	
Total	1,104 (86.7)	169 (13.3)	1,273 (100.0)	

Multivariate logistic regression for the response to the question “Will you take the COVID-19 vaccine when it becomes available” (vaccine hesitancy) as the dependent (outcome) variable and items of VAX scale, sociodemographic variables and a few other items related to influenza vaccine as the independent (predictor) variables was done, after controlling for age, education, nationality, marital, and employment status. Female gender, “I feel safe after being vaccinated (strongly disagree, somewhat disagree, not sure)”, “Covid-19 is a new disease and vaccines against it have not been fully tested and will not be safe (strongly disagree, somewhat disagree, not sure)”, “I feel protected after being vaccinated (not sure)”, and “I worry about the unknown effects of vaccines in the future (somewhat disagree, not sure)”, were significantly associated with vaccine hesitancy. The results were shown in Table 4.

**Table 4:** Multivariate logistic regression showing predictors of vaccine hesitancy.

**Predictors**	**OR (95% CI)**	***p*-value**
Female gender	0.69 (0.49–0.93)	0.016
Strongly disagree that I feel safe after being vaccinated	8.78 (3.67–20.99)	0.001
Somewhat disagree that I feel safe after being vaccinated	8.69 (3.89–19.40)	0.001
Not sure that I feel safe after being vaccinated	3.83 (1.89–7.77)	0.001
Strongly disagree that Covid-19 is a new disease and vaccines against it have not been fully tested and will not be safe	8.78 (3.67–20.99)	0.001
Somewhat disagree that Covid-19 is a new disease and vaccines against it have not been fully tested and will not be safe	0.40 (0.22–0.74)	0.003
Not sure I will feel protected after being vaccinated	3.00 (1.52–5.92)	0.002
Somewhat disagree that I worry about the unknown effects of vaccines in the future	0.39 (0.21–0.72)	0.003
Not sure that I worry about the unknown effects of vaccines in the future	0.32 (0.21–0.50)	0.001
Somewhat agree that I worry about the unknown effects of vaccines in the future	0.61 (0.41–0.92)	0.019
Strongly disagree that COVID-19 is not a disease	0.72 (0.41–1.26)	0.252
Salaried job	0.23 (0.05–1.06)	0.060
Unemployed	0.14 (0.01–1.13)	0.066

## Discussion

Our study is one of the largest surveys that addressed the attitudes toward vaccination in HCWs in the Middle East during the COVID-19 pandemic. The main finding of this study is that among the surveyed HCWs, 12.9% displayed hesitancy toward getting vaccinated with a COVID-19 vaccine, and a further 25.31% were unsure whether they would accept the vaccination or not. In a recent survey conducted among the Saudi HCWs (*n* = 736) in December 2020, using a non-standardized survey questionnaire, the vaccine hesitancy rate was reported to be 49.48% (24). Similarly, in an online survey conducted among the French HCWs between March and July 2020, the vaccine hesitancy rate reported was 25.9% (25). Another study done by Di Gennaro et al. (26) among the Italian HCWs, reported a vaccine hesitancy of 7%. The vaccine hesitancy rate of 12.9% in our study is much lower than the Saudi and French studies and slightly higher than the Italian study. This variation might be a reflection of the study methodology, the representative population, and the different healthcare systems.

In our study, age (older than 65 years) was significantly associated with vaccine hesitancy, which is in corroboration with the survey done by Schwarzinger et al. (27) where age displayed an inverted U-shaped relationship. In the same study, vaccine hesitancy was significantly higher in females than males, which is in line with our finding. A possible reason may be that females often consider the impact on their children and fertility (25). However, in contrast, in a study conducted in Saudi Arabia, the vaccine hesitancy was higher in males than females, although we don't have an explanation for this discrepancy (24). In our study, marital status has a strong influence on vaccine hesitancy, particularly among the married respondents. Previous studies also showed that marital status might affect vaccine hesitancy, with single parents or those divorced demonstrating increased vaccine hesitancy (28).

The main reason for vaccine acceptance in our survey was a better understanding of the disease and vaccines, which possibly enables the respondents to make an informed and confident decision on vaccine acceptance. Interestingly, a previous study showed that perceived susceptibility to and seriousness of a vaccine-preventable disease as an indicator of a better understanding of the disease that might lead to vaccine acceptance (25).

Recent studies showed that HCWs willing to accept the vaccine were more likely to recommend vaccines to friends, family, and patients (27–29). Similar findings were observed in our study.

In the VAX hesitancy scale, 26.3% of respondents reported negative attitudes about safety and trust about the vaccines. Furthermore, the hesitancy was also attributed to the concerns regarding safety among the children in their family, the chance of getting any unknown illness in the future, and a preference for natural over vaccine-induced immunity. Similar findings were also observed in our main survey on the general population (21) suggesting that these findings are not specifically applied to only HCWs.

Of note, on multivariate analysis, we found female gender, the perception that vaccines are not safe at the time of vaccination, a perceived lack of safety after vaccination, and doubts over vaccine protection were the significant predictors of vaccine hesitancy. Interestingly, having a chronic medical condition was not a significant predictor. To date, there are no large cohort studies available to authenticate the efficacy of COVID-19 vaccines. Therefore, the first generation may have limited efficacy, which leads to a loss of trust in the current COVID-19 vaccines (30). A previous study indicated that the safety, efficacy, and effectiveness of COVID-19 were the hallmark predictors of COVID-19 vaccine hesitancy (31).

The present study was conducted in a distinct part of the globe with diverse demographics, and the majority were migrant populations, including the HCWs. We also surveyed when COVID-19 vaccine producers reported their efficacy data and initiated mass immunization programs worldwide. Besides, we used a validated vaccine hesitancy tool, and the outcome measures were based on internationally established vaccine hesitancy parameters.

## Conclusion

Vaccine hesitancy has a significantly negative impact on a planned immunization program's successful outcome, and it has been considered a global threat to universal immunization programs. In our study, the majority of the HCWs accepted to take the COVID-19 vaccine. However, 1 in 8 HCWs was vaccine hesitant. The significant predictors of vaccine hesitancy were female gender, concerns about vaccine safety, safety after the vaccination, and doubts about the vaccine's protection. Education about the vaccine's safety and efficacy can potentially improve acceptance among healthcare workers.

## Data Availability Statement

The raw data supporting the conclusions of this article will be made available by the authors, without undue reservation.

## Ethics Statement

The studies involving human participants were reviewed and approved by Medical Research Council of the Hamad Medical Corporation. MRC approval-01-20-930. The patients/participants provided their written informed consent to participate in this study.

## Author Contributions

RK: conceptualization, data analysis, and interpretation and writing—original draft and editing. MA: conceptualization, methodology, supervision, validation, and writing—review and editing. NE: data analysis, interpretation, and writing—editing. SR: conceptualization, methodology, supervision, validation, and writing—review and editing. All authors contributed to the interpretation of the results, critically revised the paper, and agreed on the final version for submission.

## Conflict of Interest

The authors declare that the research was conducted in the absence of any commercial or financial relationships that could be construed as a potential conflict of interest.

## Publisher's Note

All claims expressed in this article are solely those of the authors and do not necessarily represent those of their affiliated organizations, or those of the publisher, the editors and the reviewers. Any product that may be evaluated in this article, or claim that may be made by its manufacturer, is not guaranteed or endorsed by the publisher.
